# Trophectoderm biopsy is associated with adverse obstetric outcomes rather than neonatal outcomes

**DOI:** 10.1186/s12884-023-05466-z

**Published:** 2023-03-04

**Authors:** Hui Ji, Mian-Qiu Zhang, Qiao Zhou, Song Zhang, Li Dong, Xiu-Ling Li, Chun Zhao, Hui Ding, Xiu-Feng Ling

**Affiliations:** Department of Reproductive Medicine, Women’s Hospital of Nanjing Medical University, Nanjing Maternity and Child Health Care Hospital, Nanjing, China

**Keywords:** Preimplantation genetic testing, Trophectoderm biopsy, Frozen-thawed blastocyst transfer, Singleton pregnancy, Obstetric outcomes, Neonatal outcomes, Propensity score matching

## Abstract

**Background:**

With the wide application of preimplantation genetic testing (PGT) with trophectoderm (TE) biopsy, the safety of PGT has always been a concern. Since TE subsequently forms the placenta, it is speculated that the removal of these cells was associated with adverse obstetrical or neonatal outcomes after single frozen-thawed blastocyst transfer (FBT). Previous studies report contradictory findings with respect to TE biopsy and obstetric and neonatal outcomes.

**Methods:**

We conducted a retrospective cohort study including 720 patients with singleton pregnancies from single FBT cycles who delivered at the same university-affiliated hospital between January 2019 and March 2022. The cohorts were divided into two groups: the PGT group (blastocysts with TE biopsy, *n* = 223) and the control group (blastocysts without biopsy, *n* = 497). The PGT group was matched with the control group by propensity score matching (PSM) analysis at a ratio of 1:2. The enrolled sample sizes in the two groups were 215 and 385, respectively.

**Results:**

Patient demographic characteristics were comparable between the groups after PSM except for the proportion of recurrent pregnancy loss, which was significantly higher in the PGT cohort (31.2 vs. 4.2%, *P* < 0.001). Patients in the PGT group had significantly higher rates of gestational hypertension (6.0 vs. 2.6%, adjusted odds ratio (aOR) 2.91, 95% confidence interval (CI) 1.18–7.18, *P* = 0.020) and abnormal umbilical cord (13.0 vs. 7.8%, aOR 1.94, 95% CI 1.08–3.48, *P* = 0.026). However, the occurrence of premature rupture of membranes (PROM) (12.1 vs. 19.7%, aOR 0.59, 95% CI 0.35–0.99, *P* = 0.047) was significantly lower in biopsied blastocysts than in unbiopsied embryos. There were no significant differences in regard to other obstetric and neonatal outcomes between the two groups.

**Conclusions:**

Trophectoderm biopsy is a safe approach, as the neonatal outcomes from biopsied and unbiopsied embryos were comparable. Furthermore, PGT is associated with higher risks of gestational hypertension and abnormal umbilical cord but may have a protective effect on PROM.

## Background

Since the first successful pregnancies following preimplantation genetic testing (PGT) reported in 1990 [1], the technology has been advancing and increasing rapidly. PGT comprises a spectrum of types, including PGT for aneuploidy (PGT-A), PGT for structural rearrangements (PGT-SR), and PGT for monogenic disorders (PGT-M). Regardless of these different indications, the key step is extracting the DNA from oocytes (polar bodies) or embryos (cleavage stage or blastocyst) for genetic testing. Compared with the cleavage stage, the current literature favors blastocyst-stage biopsy due to its enhanced precision and reduced diagnostic error [2]. Additionally, the methodology has evolved to next-generation sequencing (NGS) due to its reduced costs and enhanced precision [3]. Consequently, PGT at the blastocyst stage with the use of NGS and single blastocyst transfer in subsequent frozen-thawed transfer (FET) cycles has become common practice. Despite the emerging clinical application, there have been concerns about the safety of obstetric and neonatal outcomes following trophectoderm (TE) biopsy. The removed TE cells are destined to form the placenta, leading to the suspicion of potential risks of adverse pregnancy outcomes that are associated with abnormal placentation. Additionally, follow-up data on children conceived by PGT are paramount for evaluating the safety of this technique.

Previous studies report mixed results with respect to the effect of TE biopsy on maternal and neonatal outcomes following frozen-thawed blastocyst transfer (FBT). One recently published meta-analysis indicated that PGT pregnancies were associated with a higher risk of hypertensive disorders of pregnancy (HDP) than in vitro fertilization (IVF)/intracytoplasmic sperm injection (ICSI) pregnancies [4]. A systematic review and meta-analysis by Hou et al. [5] found inconsistent results; PGT after blastocyst biopsy and FBT did not increase the risk of HDP. Additionally, several retrospective studies have found higher risks of gestational hypertension [6] and preeclampsia [7] after transferring biopsied blastocysts. Nevertheless, some authors discovered that embryo biopsy does not increase adverse events related to placentation or obstetric conditions [8–11].

In regard to neonatal outcomes, blastocyst biopsy did not add additional risks following FET cycles [6–9, 12–14]. In contrast to these observations, pregnancies conceived after PGT were associated with an increased risk of preterm birth [15] and decreased risks of very preterm birth (VPTB) [4, 16] and very low birth weight (VLBW) [4, 5], as indicated previously.

The relevant data from previous studies mainly relied on patient reports instead of electronic medical records (EMRs), which are subject to recall bias. In such cases, some important maternal and neonatal outcomes could be missed or ignored, causing study conclusions to be less comprehensive. Prompted by the aforementioned information, this study included data from EMRs in both reproductive and obstetric departments in the same hospital. We aimed to compare obstetric and neonatal results of singleton births after single FBT cycles with and without embryo biopsy, adding more details to assess the safety of PGT technology.

## Methods

### Patients

The study was reviewed and approved by the Ethics Committee of Nanjing Maternity and Child Health Care Hospital according to the Declaration of Helsinki (2022KY-049), conducting at the reproductive center and the obstetric department of the same hospital. The delivery timing spanned from January 2019 to March 2022. The inclusion criteria were as follows: (a) single D5 or D6 blastocyst transfer; (b) first or second FET transfer; (c) autologous oocytes; and (d) singleton delivery at ≥ 28 weeks of gestation. The exclusion criteria were as follows: (a) uterine malformation; (b) delivery at other hospitals; (c) identical twins; (d) stillbirth (fetal death after 28 weeks of pregnancy); and (e) missed cycle data.

### Treatment protocol

The protocols of antagonist ovarian stimulation, embryo culture, blastocyst morphological evaluation, embryo biopsy, vitrification and thawing, endometrial preparation, and luteal phase support were introduced in our previously published articles [17]. Oocytes were inseminated by IVF or ICSI based on sperm quality, and all PGT cycles was performed by ICSI. For biopsied blastocysts (Days 5–6 after fertilization), we used a laser (Hamilton Thorne Inc., Beverly, USA) to assist in the opening of a 10–20 μm hole in the zona pellucida. After gently aspirating 4–5 TE cells, the samples were processed for NGS analysis (Illumina, San Diego, CA, USA). Only euploid embryos or mosaic embryos, following the guidance of the Preimplantation Genetics Diagnosis International Society, were transferred in later FET cycles [18]. Blastocysts greater than 3BB before vitrification were defined as good-quality embryos. FET was performed in either a natural or artificial cycle (AC) based on patient and physician preferences. In natural cycles (NCs), patients were monitored for follicular growth and triggered with a shot of 10,000 IU human chorionic gonadotrophin once the leading follicle reached 18 mm. Patients receiving the AC protocol were administered exogenous estrogen and progesterone. Luteal phase support was commenced once ovulation was confirmed in NC-FET or achieved the adequate endometrial preparation in AC-FET.

### Obstetric outcomes

All demographic data regarding FET cycles were collected from our fertility center’s EMRs. The obstetric and neonatal complications were obtained from the EMR system in the Department of Obstetrics of our hospital. Live birth was defined as a fetus born alive after 28 weeks of pregnancy. The primary aim was to examine obstetric outcomes, specifically the incidence of gestational hypertension. The four categories of HDP were preeclampsia, gestational hypertension, superimposed preeclampsia, and chronic hypertension. Gestational hypertension was defined as hypertension after 20 weeks of gestational age without proteinuria. Additional obstetric outcomes that were examined included gestational diabetes, premature rupture of membranes (PROM, beyond 37 weeks of gestation and prior to the onset of labor), preterm premature rupture of membranes (PPROM, rupture of membranes prior to 37 weeks of gestation), placenta previa, placenta accreta, abnormal umbilical cord, polyhydramnios, oligohydramnios, postpartum hemorrhage (estimated blood loss ≥ 500 mL in a vaginal delivery or ≥ 1000 mL in a cesarean delivery), and cesarean delivery. The spectrum of abnormal umbilical cord includes the following types: presentation of umbilical cord, torsion of cord, excessively long cord, true knot, and abnormal insertion (e.g., vasa previa, battledore placenta, and velamentous insertion).

### Neonatal outcomes

The secondary aim was to examine the following neonatal results: gestational age (day); preterm birth (≥ 32 to < 37 weeks’ gestational age); very preterm birth (≥ 28 to < 32 weeks’ gestational age); birth weight (grams); small for gestational age (SGA, birth weight < 10th percentile for gestational age using population-based growth curves); large for gestational age (LGA, birth weight > 90th percentile for gestational age); low birth weight (< 2500 g); macrosomia (≥ 4000 g); mild birth asphyxia (1-min Apgar score of 4–7); and birth defects as classified previously [19].

### Statistical analysis

Statistical analyses were conducted using SPSS 24.0 (IBM, NY, USA). Propensity score matching (PSM) was applied to control the characteristic differences between two different groups, minimizing potential confounders and selection bias. Variables such as maternal age, body mass index (BMI), parity, endometrial preparation regimen, endometrial thickness (EMT), embryo developmental stage, and good-quality embryos were included. Patients in the PGT group were matched in a 1:2 ratio to those in the control group using nearest-neighbor matching with a 0.1 caliper radius.

Normally distributed continuous data are presented as mean (standard deviation), and Student’s t test was used to compare differences within groups. Chi square or Fisher’s exact tests were used for categorical data, which are presented as frequencies and percentages. Multivariable logistic regression was applied to evaluate the effect of PGT technology on different obstetric and neonatal complications. Effect estimates are presented as adjusted odds ratios (aORs) with 95% confidence intervals (CIs). *P* < 0.05 was considered statistically significant.

## Results

**Fig. 1:**
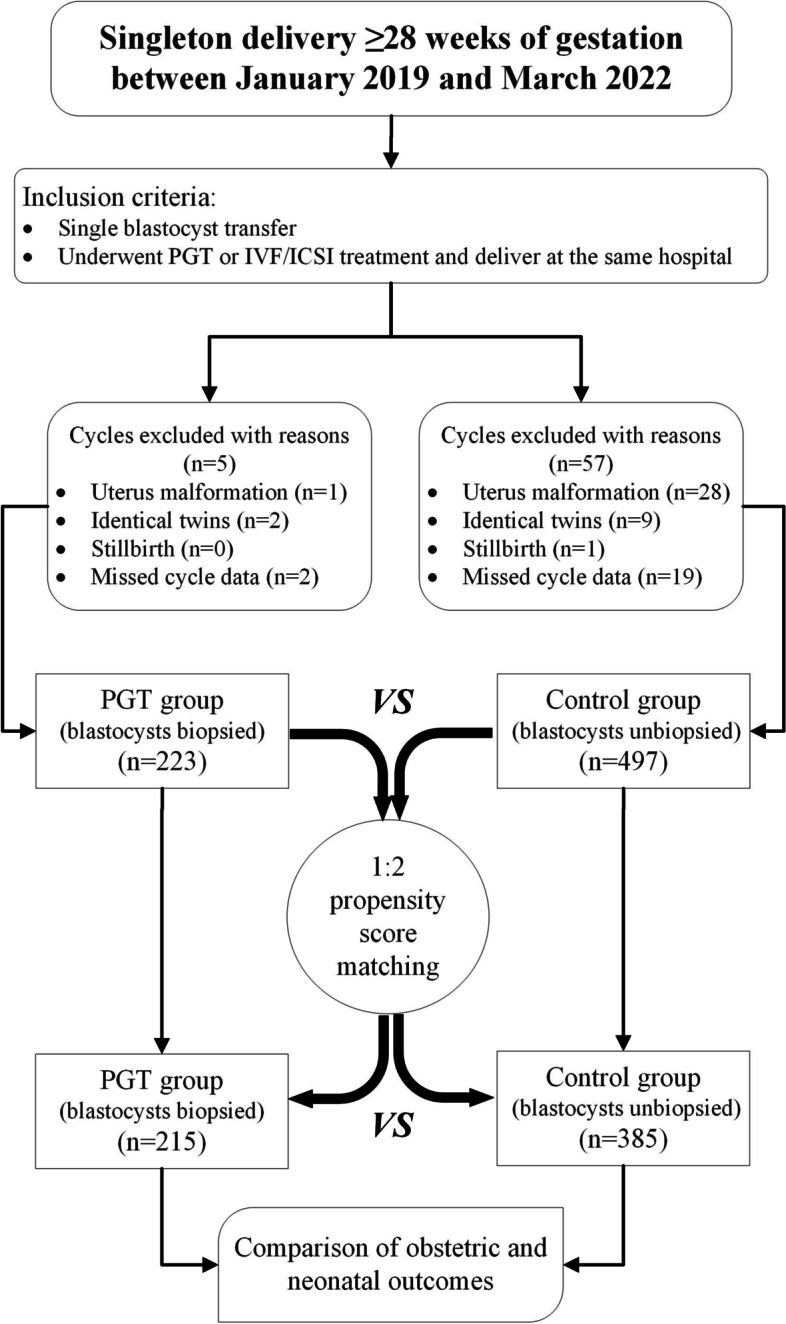
Flow chart

Figure 1 shows the study inclusion and exclusion criteria. According to whether PGT technology was applied, we divided the patients into two groups: the PGT group and the control group. A total of 720 cycles were eventually enrolled, including 223 PGT and 497 control cycles. The sample sizes for the two groups after PSM were 215 and 385, respectively. As shown in Table 1, the EMT and the proportion of recurrent pregnancy loss (RPL), endometrial preparation, embryo developmental stage and good-quality embryo transfer cycles were statistically significant between the two groups in the initial estimation (*P* < 0.05 for all). Due to the immense discrepancy of RPL within the groups, this covariate was not included in the final PSM model. All the parameters depicted no significant difference within groups after PSM analysis except for the RPL proportion. Subjects who underwent PGT-FET had a significantly higher prevalence of RPL than those who underwent FET alone (31.2 vs. 4.2%, *P* < 0.001).

**Table 1:** Baseline characteristics before and after propensity score matching

**Characteristic**	**Before PSM**			**After PSM**		
	**PGT group**	**Control group**	***P* value**	**PGT group**	**Control group**	***P* value**
	**(*n* = 223)**	**(*n* = 497)**		**(*n* = 215)**	**(*n* = 385)**	
Maternal age (years)	31.1 ± 4.3	31.1 ± 3.8	0.921	31.1 ± 4.3	31.1 ± 3.8	0.965
BMI (kg/m^2^)	22.2 ± 2.9	22.0 ± 3.1	0.272	22.1 ± 2.7	22.0 ± 3.2	0.778
Maternal parity (nulliparous)	173 (77.6)	410 (82.5)	0.120	165 (76.7)	301 (78.2)	0.685
Paternal age (years)	32.7 ± 5.8	32.8 ± 5.1	0.783	32.8 ± 5.8	32.7 ± 4.9	0.857
Duration of infertility, n (%)			0.131			0.356
1–2	136 (61.0)	273 (54.9)		131 (60.9)	220 (57.1)	
3–5	59 (26.5)	169 (34.0)		58 (27.0)	125 (32.5)	
≥ 6	28 (12.6)	55 (11.1)		26 (12.1)	40 (10.4)	
Cause of infertility, n (%)			0.114			0.180
Female	115 (51.6)	269 (54.1)		113 (52.6)	205 (53.2)	
Male	21 (9.4)	24 (4.8)		20 (9.3)	19 (4.9)	
Combined	82 (36.8)	196 (39.4)		77 (35.8)	154 (40.0)	
Unknown	5 (2.2)	8 (1.6)		5 (2.3)	7 (1.8)	
Reason of PGT, n (%)						
PGT-A	138 (61.9)	/		133 (61.9)	/	
PGT-SR	73 (32.7)	/		70 (32.6)	/	
PGT-M	12 (5.4)	/		12 (5.6)	/	
Previous history						
Smoking	0 (0.0)	3 (0.6)	0.556	0 (0.0)	2 (0.5)	0.539
PCOS	22 (9.9)	70 (14.1)	0.117	21 (9.8)	54 (14.0)	0.130
Diabetes	0 (0.0)	2 (0.4)	1.000	0 (0.0)	1 (0.3)	1.000
Hypertension	1 (0.4)	3 (0.6)	1.000	1 (0.5)	2 (0.5)	1.000
Thyroid dysfunction	23 (10.3)	35 (7.0)	0.136	23 (10.7)	27 (7.0)	0.117
Autoimmune disease	6 (2.7)	8 (1.6)	0.383	6 (2.8)	6 (1.6)	0.365
Recurrent pregnancy loss	68 (30.5)	19 (3.8)	< 0.001	67 (31.2)	16 (4.2)	< 0.001
Repeated implantation failure	2 (0.9)	1 (0.2)	0.228	2 (0.9)	1 (0.3)	0.293
Hormone levels						
Basal FSH (mIU/mL)	7.6 ± 2.1	7.7 ± 2.3	0.848	7.6 ± 2.1	7.6 ± 2.3	0.892
Basal LH (mIU/mL)	5.1 ± 3.0	5.3 ± 3.2	0.479	5.1 ± 3.0	5.4 ± 3.3	0.310
Basal E_2_ (pg/mL)	44.9 ± 23.8	46.7 ± 26.7	0.380	45.0 ± 23.9	47.7 ± 28.3	0.229
AMH (ng/mL)	5.5 ± 4.4	5.6 ± 4.2	0.701	5.6 ± 4.4	5.8 ± 4.4	0.509
Cycle rank, n (%)			0.385			0.301
1	148 (66.4)	346 (69.6)		142 (66.1)	270 (70.1)	
2	75 (33.6)	151 (30.4)		73 (34.0)	115 (29.9)	
Endometrial preparation, n (%)			0.020			0.893
NC	47 (21.1)	146 (29.4)		47 (21.9)	86 (22.3)	
AC	176 (78.9)	351 (70.6)		168 (78.1)	299 (77.7)	
Endometrial thickness (mm)	9.0 ± 1.5	9.5 ± 1.8	< 0.001	9.0 ± 1.5	9.2 ± 1.5	0.203
Embryo developmental stage, n (%)			0.001			0.151
D5	121 (54.3)	336 (67.6)		120 (55.8)	238 (61.8)	
D6	102 (45.7)	161 (32.4)		95 (44.2)	134 (38.2)	
Good-quality embryo, n (%)	161 (72.2)	401 (80.7)	0.011	161 (74.9)	300 (77.9)	0.398

Abbreviation: *PGT* preimplantation genetic testing, *PSM* propensity score matching, *BMI* body mass index, *PGT-A* preimplantation genetic testing for aneuploidy, *PGT-SR* preimplantation genetic testing for structural rearrangements, *PGT-M* preimplantation genetic testing for monogenic disorders, *PCOS* polycystic ovarian syndrome, *FSH* follicle-stimulating hormone, *LH* luteinizing hormone, *E*_*2*_ estradiol, *AMH* anti-Müllerian hormone, *NC* natural cycle, AC artificial cycle. A good-quality embryo was day 5 or 6 blastocyst grading 3BB or higher (AA, AB, BA, BB) before vitrification according to the grading scale proposed by Gardner. Data are presented as mean ± SD or n (%)

Concerning obstetric outcomes, the PGT group had significantly higher incidences of gestational hypertension (6.0 vs. 2.6%, *P* = 0.035) and abnormal umbilical cord (13.0 vs. 7.8%, *P* = 0.038) and a significantly lower rate of PROM (12.1 vs. 19.7%, *P* = 0.017) than the control group. Moreover, there was a slightly increased rate of HDP in the PGT group compared with the control group (13.5 vs. 8.8%, *P* = 0.074). The two groups showed comparable rates of preeclampsia, gestational diabetes, PPROM, placenta previa, placenta accreta, placenta abruption, polyhydramnios, oligohydramnios, postpartum hemorrhage, and cesarean delivery (Table 2).

**Table 2:** Obstetric and neonatal outcomes of singleton live births of groups with and without trophectoderm biopsy

Characteristic	PGT group	Control group	*P* value
	**(*n* = 215)**	**(*n* = 385)**	
**Obstetric outcomes**			
HDP, n (%)	29 (13.5)	34 (8.8)	0.074
Gestational hypertension, n (%)	13 (6.0)	10 (2.6)	0.035
Preeclampsia, n (%)	13 (6.0)	18 (4.7)	0.467
Gestational diabetes, n (%)	77 (35.8)	119 (30.9)	0.219
PROM, n (%)	26 (12.1)	76 (19.7)	0.017
PPROM, n (%)	5 (2.3)	20 (5.2)	0.092
Placenta previa, n (%)	6 (2.8)	19 (4.9)	0.208
Placenta accreta, n (%)	6 (2.8)	14 (3.6)	0.580
Placenta abruption, n (%)	1 (0.5)	1 (0.3)	1.000
Abnormal umbilical cord, n (%)	28 (13.0)	30 (7.8)	0.038
Polyhydramnios, n (%)	6 (2.8)	12 (3.1)	0.822
Oligohydramnios, n (%)	5 (2.3)	7 (1.8)	0.763
postpartum hemorrhage, n (%)	28 (13.0)	61 (15.8)	0.351
Cesarean delivery	176 (81.9)	309 (80.3)	0.633
**Neonatal outcomes**			
Gestational age (day)	271.5 ± 9.6	270.8 ± 13.4	0.429
Preterm birth, n (%)	14 (6.5)	40 (10.4)	0.111
Very preterm birth, n (%)	1 (0.5)	8 (2.1)	0.168
Birth weight (g)	3383.6 ± 475.9	3356.6 ± 526.3	0.533
SGA, n (%)	6 (2.8)	11 (2.9)	0.962
LGA, n (%)	51 (23.7)	105 (27.3)	0.342
Low birth weight, n (%)	10 (4.7)	18 (4.7)	0.989
Macrosomia, n (%)	19 (8.8)	31 (8.1)	0.739
Mild birth asphyxia, n (%)	4 (1.9)	3 (0.8)	0.257
Birth defect, n (%)	1 (0.5)	3 (0.8)	1.000

Abbreviation: *HDP* hypertensive disorders of pregnancy, *PROM* premature rupture of membranes, *PPROM* preterm premature rupture of membranes, *SGA* small for gestational age, *LGA* large for gestational age

Table 2 also demonstrates the comparison of neonatal outcomes. There were no significant differences in terms of preterm birth, VPTB, SGA, LGA, low birth weight, macrosomia, mild asphyxia, or birth defects (*P* > 0.05). Additionally, the gestational age and birth weight in the two groups did not differ statistically.

To predict the effect of TE biopsy on obstetric complications with *P* value < 0.1 in the univariate analysis, multiple logistic regression analysis was performed, incorporating female age, BMI, parity (1 vs. 0), RPL (yes vs. no), endometrial preparation protocol (AC vs. NC), EMT, and good-quality embryos (yes vs. no) (Table 3). The PGT method had significant positive influences on gestational hypertension (aOR 2.91, 95% CI 1.18–7.18, *P* = 0.020) and abnormal umbilical cord (aOR 1.94, 95% CI 1.08–3.48, *P* = 0.026). The risk of PROM remained lower with PGT singleton pregnancies than with IVF/ICSI singleton pregnancies (aOR 0.59, 95% CI 0.35–0.99, *P* = 0.047). Although applying PGT seemed to increase the HDP rate and decrease the PPROM rate, it did not reach statistical significance (aOR 1.64, 95% CI 0.91–2.95, *P* = 0.103 for HDP; aOR 0.50, 95% CI 0.18–1.42, *P* = 0.193 for PPROM).

**Table 3:** The effect of trophectoderm biopsy on obstetric complications

	aOR	95% CI	*P* value
HDP	1.64	0.91–2.95	0.103
Gestational hypertension	2.91	1.18–7.18	0.020
PROM	0.59	0.35–0.99	0.047
PPROM	0.50	0.18–1.42	0.193
Abnormal umbilical cord	1.94	1.08–3.48	0.026

Abbreviation: *aOR* adjusted odds ratio, *CI* confidence interval. The enrolled obstetric complications were those with a *P*-value < 0.1 in the univariate analysis. Multivariable logistic regression analysis adjusted for female age, BMI, parity (1 vs. 0), RPL (yes vs. no), endometrial preparation protocol (AC vs. NC), endometrial thickness, and good-quality embryos (yes vs. no)

## Discussion

To date, it is still unclear whether TE biopsy poses additional obstetric and neonatal risks compared with pregnancies conceived by IVF/ICSI without biopsy. Several studies have reported inconsistent results in singleton pregnancies following PGT application. The present study suggested that embryo biopsy may increase the risks of gestational hypertension and abnormal umbilical cord, but may result in a significantly lower rate of PROM. Furthermore, no statistically significant differences in adverse neonatal outcomes were found in our analysis, providing reassurance toward expanding the utilization of PGT in clinical practice.

The present study was in line with previous articles reporting that TE biopsy was related to placentation disorders [4, 6, 7, 14]. The risk of preeclampsia from our data showed no significant difference between biopsied embryos and unbiopsied embryos. The study by Zhang et al. [7] found a three-fold increase in the odds of preeclampsia in singleton live births following PGT cycles. Although they limited the analysis to FBT cycles and data were collected from hospital EMRs, one key difference between their study and ours is the different sample sizes. Their study enrolled only 134 PGT cycles and 124 FET cycles without PGT. Alternatively, our results were consistent with a recent study consisting of 214 PGT singleton pregnancies and 617 IVF/ICSI singleton pregnancies [6]. The rate of gestational hypertension was higher in the PGT cohort, while the preeclampsia incidence revealed no difference. The detrimental impact on placental function was mainly due to the TE biopsy procedure, in which the removed TE cells were destined to form the placenta. An animal study performed blastomere biopsy at the cleavage stage and observed increased placental oxidative stress and inflammation, suggesting that placental development was vulnerable after blastomere biopsy procedures [20]. Embryo gene transcription in a mouse model was altered after blastocyst biopsy [21]; plausible mechanisms may exert similar consequences in human offspring following PGT treatment. Furthermore, the placenta is crucial for fetal growth, as it provides nutrients, secretes hormones, and regulates immunity. The extraction of several TE cells may directly impair placental formation and function [4, 6].

Most notably, our data demonstrated a significantly decreased risk of PROM in singleton pregnancies conceived from biopsied blastocysts in FET cycles. The topic of PPROM has been of constant interest in the literature. Zhang et al. [7] found a decreased odds of PPROM in the PGT group, but the difference was not significant (7.0 vs. 11.6%, aOR 0.63, 95% CI 0.28–1.42, *P* = 1.0). Moreover, other previous studies revealed comparable risks regarding PPROM [4, 5, 7, 14, 22, 23]. However, there is a paucity of available data investigating maternal outcomes of PROM in PGT-induced pregnancies. Here, we demonstrated a significantly lower rate of PROM in the PGT group after 37 weeks of gestation and before the onset of labor (12.1 vs. 19.7%, aOR 0.59, 95% CI 0.35–0.99, *P* = 0.047). Currently, spontaneous rupture of the fetal membranes after full term is considerably safer than that before 37 weeks of gestation. Although the incidence was significantly higher in patients who underwent IVF/ICSI, PGT did not increase adverse maternal and neonatal outcomes. The mechanism by which PGT decreases the PROM rate is unclear. The resilience of fetal membranes to rupture is vitally important to maintain the integrity of the amniotic cavity and a successful pregnancy outcome. Trophoblasts are an important component of the fetal membrane and chorion leave, as the latter two are made up of fetal membranes. We speculated that TE biopsy could alter the tensile strength of the fetal membranes. Overall, because the available studies are scant, more studies are warranted to validate the effect of PGT on the prevalence of PROM.

Previous publications confirmed that vasa previa [24–27] and abnormal cord insertion [27–29] are more frequent in pregnancies conceived by assisted reproductive technology. Nonetheless, no study has further compared the odds of abnormal umbilical cord after PGT technology versus IVF/ICSI technology. To the best of our knowledge, the current study was the first to explore whether PGT is associated with an increased rate of umbilical cord abnormalities compared with IVF/ICSI. Instead of comparing each type of aberrant umbilical cord, our study analyzed the overall rate of abnormalities due to fewer positive cases. Caution should be taken when extrapolating the conclusion to each abnormal umbilical cord category. Similarly, the underlying mechanism has not been fully elucidated. Since the umbilical cord is a crucial part of the placenta, we hypothesized that its formation and function may be adversely affected by TE biopsy. Further studies are required to characterize TE biopsy as a risk factor for umbilical cord abnormalities.

With regard to neonatal outcomes, the current study was consistent with most of the previous articles [4, 6–10, 13, 14, 30, 31] in which no association of adverse neonatal outcomes was found between PGT pregnancies and IVF/ICSI pregnancies. In contrast, a large cohort study contained 58,812 FET cycles and 59,120 PGT cycles from the American SART system [16]. They found that PGT significantly decreased the odds of VPTB (1.5% vs 1.9%, *P* = 0.0002). In our study, the rates of VPTB were 0.5% (1/215) and 2.1% (8/385) in the PGT and IVF/ICSI cohorts, respectively. Similarly, Sunkara et al. confirmed that the VPTB incidence was 0.9% (4/439) and 1.7% (1512/87571) in PGT and IVF/ICSI patients, respectively [30]. The main reason for the difference in the final conclusion was the obviously large sample size in the study by Ying et al. [16], which may turn the slight difference into statistical significance. Nevertheless, a major limitation of their study was the lack of several important factors, such as patient BMI, infertility etiology, parity, EMT, and endometrial preparation regimen. The unavailability of crucial baseline characteristics may render the conclusion credible. Moreover, two recent systematic reviews and meta-analyses suggested that PGT with TE biopsies was associated with a lower rate of VLBW [4, 5]. These two studies are difficult to compare to our study, as they included studies with donor oocyte cycles, cleavage-stage biopsies, and fresh embryo transfers. Considering the major flaws of the abovementioned papers, the current literature provides reassuring and promising results to support the safety of PGT methodology for neonatal outcomes.

Our study has several strengths and a few limitations. The strengths include the comprehensive and detailed data regarding patient information and maternal and neonatal outcomes. Since data were collected from reproductive and obstetrics departments in the same hospital, some importation outcomes were fully collected and analyzed. Unlike our research, other studies relied exclusively on patient reports, and we suspected that patients would underreport outcomes. The present study was the first to reveal a significantly lower rate of PROM and a higher rate of umbilical cord abnormalities in PGT-induced singleton pregnancies. The maternal outcomes of PROM and abnormal umbilical cord are usually neglected in the literature. Notwithstanding, this adverse obstetric outcome could inevitably pose potential risks to both mothers and their children. For instance, an aberrant condition of the umbilical cord may have detrimental influences on the fetus, such as fetal growth restriction, preterm birth, and stillbirth [32–35]. To that extent, it is worthy of close attention in further studies.

Additionally, PSM analysis was used to reduce bias to create comparable comparisons within groups. We also included some crucial confounders in the final analysis, including endometrial preparation, EMT, and embryo quality. For example, the AC protocol, compared with the NC-FET protocol, was a significant risk factor for HDP [36–38]. Apart from endometrial preparation, previous studies indicated that a thin endometrium was correlated with a decreased singleton birth weight [39] and an increase in preterm birth and intrauterine growth restriction [40]. Evidence in some studies indicated that a poor-quality single-blastocyst transfer was related to a higher risk of low birthweight, preterm birth, and congenital malformation [41–43], indicating that embryo morphological grading could make a notable difference in offspring health. Generally, specific data regarding differences in obstetric and neonatal outcomes between singleton pregnancies after TE-biopsied embryos and unbiopsied embryos are scarce. Therefore, our study could provide more valid evidence to inform both clinicians and patients.

Limitations of this study included the relatively small sample size from a single hospital. Considering its retrospective design, selection bias could not be totally excluded. Meanwhile, the heavy medical expenses of PGT treatment are borne by couples and their families in mainland China, leading to the fact that only patients with medical indications are advised to choose the PGT cycle. Thus, there was a stark difference in terms of RPL proportion between the two groups. We then employed multivariate regression analysis to minimize the obscure and possible impact of RPL on obstetrical outcomes, hoping to improve conclusion validity. Additionally, little is known about the underlying mechanism of TE biopsy associated with a lower rate of PROM and a higher odds of umbilical cord abnormalities. Applying these results should be done with caution due to some existing flaws in the current study.

## Conclusion

When compared with singleton pregnancies following a single unbiopsied blastocyst transfer, trophectoderm biopsy increases the risks of gestational hypertension and umbilical cord abnormalities but decreases the odds of PROM in FET cycles. It is also important to note that blastocyst biopsy might not exert additional complications on neonatal outcomes. Before definitive conclusions are drawn, basic scientific research and future studies with large sample sizes are warranted to confirm our results.

## Data Availability

The data generated and analyzed in this study will be availed upon request by the corresponding author.
